# Danggui Sini decoction alleviates oxaliplatin-induced peripheral neuropathy by regulating gut microbiota and potentially relieving neuroinflammation related metabolic disorder

**DOI:** 10.1186/s13020-024-00929-7

**Published:** 2024-04-07

**Authors:** Chen Chen, Jian-Lin Xu, Zhan-Cheng Gu, Shan-Shan Zhou, Guo-Li Wei, Jia-Lin Gu, Hai-Long Ma, Yan-Qi Feng, Zi-Wei Song, Zhan-Peng Yan, Shan Deng, Rong Ding, Song-Lin Li, Jie-Ge Huo

**Affiliations:** 1 Department of Oncology, Yancheng TCM Hospital Affiliated to Nanjing University of Chinese Medicine, Yancheng, 224001 Jiangsu China; 2 Department of Oncology, Yancheng TCM Hospital, Yancheng, 224001 Jiangsu China; 3 The Third Clinical Medical College, Nanjing University of Chinese Medicine, Nanjing, 210023 Jiangsu China; 4 Department of Oncology, Kunshan Hospital of Traditional Chinese Medicine, Suzhou, 215399 China; 5 Department of Pharmaceutical Analysis, Affiliated Hospital of Integrated Traditional Chinese and Western Medicine, Nanjing University of Chinese Medicine, No. 100 Shizi Street Hongshan Road, Nanjing, 210028 Jiangsu China; 6 Department of Metabolomics, Jiangsu Province Academy of Traditional Chinese Medicine, Nanjing, 210028 Jiangsu China; 7 Department of Oncology, Affiliated Hospital of Integrated Traditional Chinese and Western Medicine, Nanjing University of Chinese Medicine, No. 100 Shizi Street Hongshan Road, Nanjing, 210028 Jiangsu China; 8 Department of Oncology, Jiangsu Province Academy of Traditional Chinese Medicine, Nanjing, 210028 Jiangsu China; 9 Department of Oncology, Nanjing Lishui District Hospital of Traditional Chinese Medicine, Nanjing, 211299 Jiangsu China; 10 Department of Paediatrics, Yancheng TCM Hospital Affiliated to Nanjing University of Chinese Medicine, Yancheng, 224001 Jiangsu China; 11 Clinical Research Department of Chinese and Western Medicine, Jiangsu Province Academy of Traditional Chinese Medicine, Nanjing, 210028 Jiangsu China

**Keywords:** Danggui Sini decoction, Gut microbiota, Oxaliplatin-induced peripheral neuropathy, Neuroinflammation, Metabolic disorder, Fecal microbiota transplantation

## Abstract

**Background:**

Danggui Sini decoction (DSD), a traditional Chinese medicine formula, has the function of nourishing blood, warming meridians, and unblocking collaterals. Our clinical and animal studies had shown that DSD can effectively protect against oxaliplatin (OXA)-induced peripheral neuropathy (OIPN), but the detailed mechanisms remain uncertain. Multiple studies have confirmed that gut microbiota plays a crucial role in the development of OIPN. In this study, the potential mechanism of protective effect of DSD against OIPN by regulating gut microbiota was investigated.

**Methods:**

The neuroprotective effects of DSD against OIPN were examined on a rat model of OIPN by determining mechanical allodynia, biological features of dorsal root ganglia (DRG) as well as proinflammatory indicators. Gut microbiota dysbiosis was characterized using 16S rDNA gene sequencing and metabolism disorders were evaluated using untargeted and targeted metabolomics. Moreover the gut microbiota mediated mechanisms were validated by antibiotic intervention and fecal microbiota transplantation.

**Results:**

DSD treatment significantly alleviated OIPN symptoms by relieving mechanical allodynia, preserving DRG integrity and reducing proinflammatory indicators lipopolysaccharide (LPS), IL-6 and TNF-α. Besides, DSD restored OXA induced intestinal barrier disruption, gut microbiota dysbiosis as well as systemic metabolic disorders. Correlation analysis revealed that DSD increased bacterial genera such as *Faecalibaculum*, *Allobaculum*, *Dubosiella* and *Rhodospirillales_unclassified* were closely associated with neuroinflammation related metabolites, including positively with short-chain fatty acids (SCFAs) and sphingomyelin (d18:1/16:0), and negatively with pi-methylimidazoleacetic acid, l-glutamine and homovanillic acid. Meanwhile, antibiotic intervention apparently relieved OIPN symptoms. Furthermore, fecal microbiota transplantation further confirmed the mediated effects of gut microbiota.

**Conclusion:**

DSD alleviates OIPN by regulating gut microbiota and potentially relieving neuroinflammation related metabolic disorder.

**Supplementary Information:**

The online version contains supplementary material available at 10.1186/s13020-024-00929-7.

## Introduction

Danggui Sini decoction (DSD) is an herbal formula recorded in the “Treatise on Febrile Diseases” that consists of Angelicae Sinensis Radix from *Angelica sinensis* (Oliv.) Diels, Paeoniae Radix Alba from *Paeonia lactiflora* Pall., Cinnamomi Ramulus from *Cinnamomum cassia* Presl, Asari Radix et Rhizoma from *Asarum heterotropoides* Fr. Schmidt var. *mandshuricum* (Maxim.) Kitag., Tetrapanacis Medulla from *Tetrapanax papyrifer* (Hook.) KKoch, Jujubae Fructus from *Ziziphus jujuba* Mill. and Glycyrrhizae Radix et Rhizoma from *Glycyrrhiza uralensis* Fisch.. This prescription has the function of nourishing blood, warming meridians, and unblocking collaterals [1]. Therefore, DSD has anti-inflammatory and analgesic effects, and improves peripheral blood circulation and nerve conduction velocity [2, 3], which can mainly be used to treat diabetes-induced peripheral neuropathy, rheumatoid arthritis, and spinal cord injury-induced neuropathy.

Oxaliplatin (OXA) is a third-generation platinum-based chemotherapeutic drug that has no cross-resistance with cisplatin, and has been widely used in tumor therapy. OXA-induced peripheral neuropathy (OIPN) is the main dose-limiting adverse effect of OXA regimen, which may cause an insufficient dosage resulting in poor chemotherapy effects and severely decrease patient's quality of life [4]. When the cumulative dosage of OXA exceeds 780–850 mg/m^2^, the incidence of OIPN can reach 76–90% [5, 6] and last for several years [7, 8]. Although numerous preventive therapies are proposed to address OIPN, but many strategies are still ineffective. Duloxetine is the only drug moderately recommended by the American Society of Clinical Oncology (ASCO) for the prevention of OIPN. While this recommendation may help ameliorate the symptom of neuropathic pain in patients experiencing OIPN, but it may also cause adverse events (including weight loss, anorexia, nausea, fatigue, headaches) and weaken the cancer treatment [9, 10]. Given this controversy, identifying safe and effective therapies against OIPN is a prominent concern.

Researchers have shown OXA can cause nervous system inflammation by disrupting dysfunction of gut microbiota including intestinal permeability and bacterial metabolism, then leading to OIPN [11–13]. Gut microbiota sends signals to the nervous system by producing inflammation related metabolites and neuroactive metabolites [14]. For example, lipopolysaccharide (LPS) released by pathogenic bacteria activates toll-like receptor (TLR) signaling (especially TLR4) and produces neuroinflammation [11, 15]. While short-chain fatty acids (SCFAs) generated by beneficial bacteria improve neuroinflammation [16]. Besides, unbalanced neurotransmission can induce nervous system inflammation by triggering the release of proinflammatory cytokine [17]. Gut microbiota imbalance caused by OXA can promote the production of bacterial endotoxin (eg. LPS) [11, 18] and neurotransmitters (eg. gamma aminobutyric acid, GABA and glutamate) [19]. These will ultimately result in inflammation of the dorsal root ganglia (DRG), a main site of platinum accumulation during OXA exposure [20]. In this way, gut microbiota-mediated DRG inflammation may induce the development of OIPN [11].

The main clinical manifestations of OIPN are numbness in the hands and feet, abnormal or dull sensation, and with or without pain. It has the characteristic of worsening when encountering cold and slowing down when encountering heat, which is equivalent to the category of “Arthromyodynia” in traditional Chinese medicine. In clinical practice, the classic formula DSD could treat “Arthromyodynia” referred to the traditional function of nourishing blood, warming meridians, and unblocking collaterals [3], therefore, be used to prevent OIPN. Our previous clinical study had demonstrated convincingly that DSD could effectively prevent and treat OIPN [21]. Meanwhile, animal studies also showed that DSD had neuroprotective effects against OIPN by reducing the current amplitude of DRG cells undergoing agonists stimuli, inhibiting the inflammatory response, enhancing amounts of Nissl bodies, and improving ultra-microstructures in DRG cells [22]. Moreover, high-dose of DSD provided the best protective effect by significantly enhancing amounts of Nissl bodies, improving ultra-microstructures in DRG cells [22], and relieving mechanical allodynia in OIPN model rats than the low-dose and medium-dose of DSD [23]. However, the underlying mechanisms of DSD-mediated protection on OIPN are still unclear.

In this study, a rat model of OIPN was established to verify the protective effects of DSD. Then gut microbiota diversity and metabolic changes in feces and plasma were assessed respectively. The mediated mechanism of gut microbiota in protective effects of DSD against OIPN was further verified by fecal microbiota transplantation (FMT). Finally, the correlation among gut microbiota and metabolism involved in DSD treatment was discussed.

## Material and methods

### OXA, DSD formulation, and antibiotics

OXA was obtained from Jiangsu Hengrui Pharmaceutical Co., Ltd. (Lianyungang, China). The granules of Angelica Sinensis Radix (batch no. 211018303), Paeoniae Radix Alba (batch no. 21071953), Cinnamoni Cortex (batch no. 21100113), Asari Radix et Rhizoma (batch no. 21070893), Akebiae Caulis (batch no. 21012303), Jujubae Fructus (batch no. 21063633) and Glycyrrhizae Radix et Rhizoma (batch no. 21080543) were purchased from Tianjiang Pharmaceutical Co., Ltd. (Jiangyin, China). Antibiotics, including ampicillin (RH279015), metronidazole (RH298141), neomycin (F0649), and vancomycin (FY20390), were from Nantong Jingwei Biotechnology Co., Ltd. (Nantong, China).

### Preparation and chemical characterization of DSD

DSD was prepared according to the adult prescription raw drug dosage of 54 g: Angelica Sinensis Radix (12 g), Paeoniae Radix Alba (9 g), Cinnamomi Ramulus (9 g), Asari Radix et Rhizoma (3 g), Tetrapanacis Medulla (6 g), Jujubae Fructus (9 g), and Glycyrrhizae Radix et Rhizoma (6 g). Formula granules were dissolved in warm water at the desired volume. The final concentrations prepared are that low-dosage is 0.62 g/mL, medium-dosage is 1.24 dosage g/mL, and high-dosage is 2.48 g/mL [22]. Our previous studies showed that high-dose of DSD provided the best treatment effect [22, 23], so only a high-dose (2.48 g/mL) experimental group was established. The major chemical constituents in the DSD formula granules were qualitatively characterized by ultraperformance liquid chromatography with quadrupole time-of-flight tandem mass spectrometry (UPLC-QTOF-MS/MS) as previously described [24]. Nine main constituents, namely albiflorin, paeoniflorin, liquiritin apposite, liquiritin, galloylpaeoniflorin or galloylalbiflroin or their isomers, glyyunnanprosapogenin, glycyrrhizic acid, uralsaponin B, and ligustilide, were determined. Representative chromatograms and results of chemical marker analysis were shown in Additional file 1: Fig. S1 and Table S1. The voucher specimens were conserved in the Department of Metabolomics, Jiangsu Province Academy of Traditional Chinese Medicine.

### Animals

Male Wistar rats (weight 200 ± 20 g) were obtained from the Shanghai Municipal Institute of Family Planning (Shanghai, China). The rats were maintained under a 12-h light/dark cycle with free access to water and chow. Experimental schemes were approved by the Animal Ethics Committee of Jiangsu Province Academy of Traditional Chinese Medicine and implemented in strict accordance with the Guide for Care and Use of Laboratory Animals published by the US National Institutes of Health.

### OIPN model and experimental protocols

**Fig. 1:**
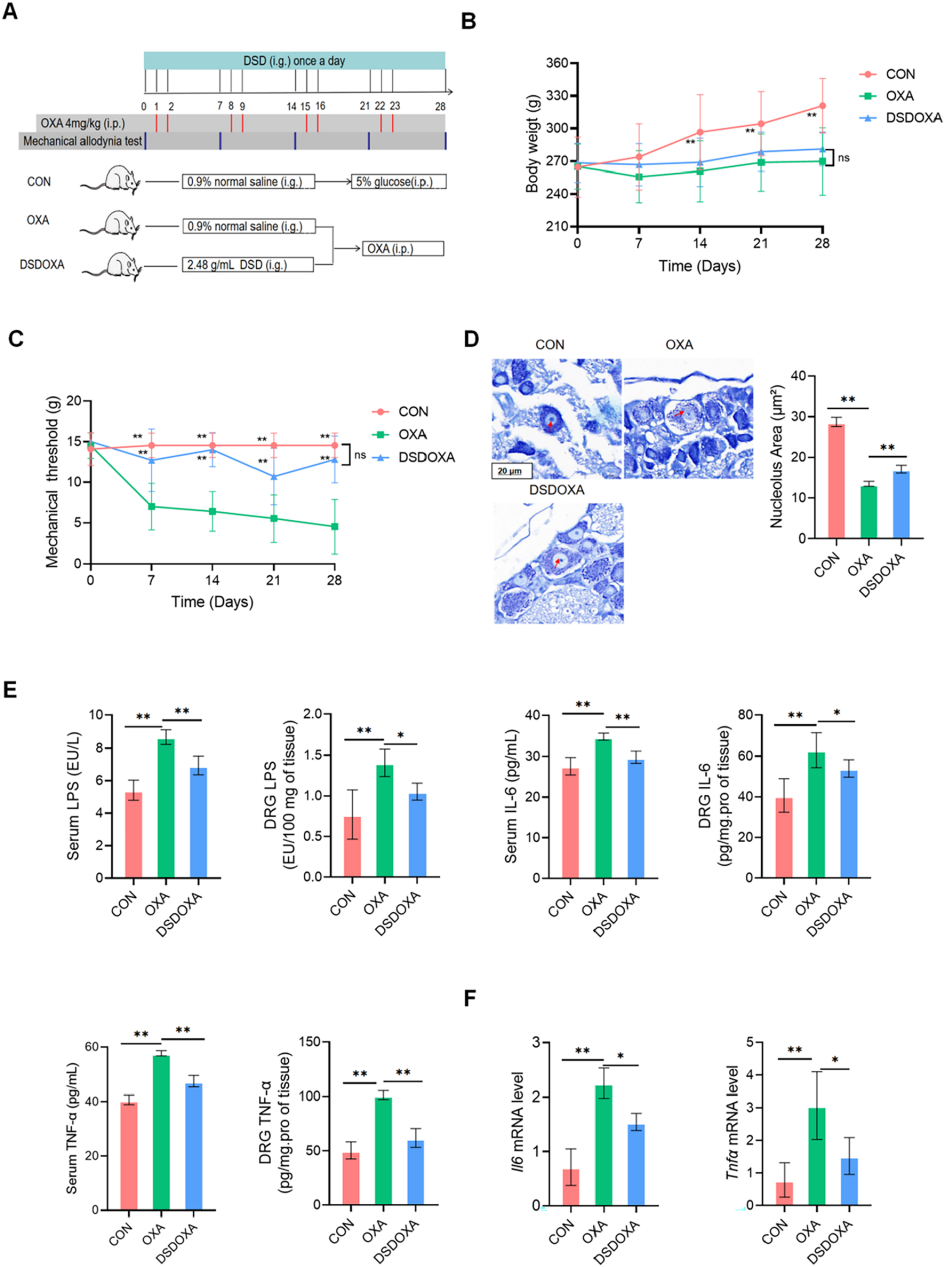
DSD alleviated OIPN and the inflammatory response. **A** Protocols of drug administration and mechanical allodynia test, in the rat OIPN model. **B** Body weight measurements. **C** Measurement of withdrawal threshold for mechanical allodynia (von Frey test). **D** Representative images of Nissl-stained DRG sections (63 ×). Lightly stained cytoplasm and shrunken nucleoli (red arrow) were analyzed. Histograms showed nucleolar area. **E** ELISA-based detection of LPS, IL-6, and TNF-α in serum and DRG extracts. **F** Analysis of IL-6 and TNF-α mRNA levels in DRG by qPCR. *P < 0.05, **P < 0.01, vs. OXA group; n = 11 in the CON group; n = 10 in the OXA and DSDOXA groups, n = 9 in the ABXOXA group

Rats were randomly divided into four groups (n = 12 per group): Control (CON), Oxaliplatin (OXA), DSD_Oxaliplatin (DSDOXA), and ABX_Oxaliplatin (ABXOXA). We established the OIPN model based on a previously described method [25]. In brief, lyophilized OXA dissolved in a 5% glucose solution was intraperitoneally injected into rats (4 mg/kg twice per week for 4 weeks) on days 1, 2, 8, 9, 15, 16, 22, and 23. The antibiotic cocktail (ABX) was composed of 1 g/L of ampicillin, neomycin, metronidazole and 0.5 g/L of vancomycin [26], and was freshly prepared per two days and kept wrapped in tin foil to avoid exposure to light. The CON group was left untreated, while OIPN was induced in the other three groups as described above. DSD was orally administered to rats in the DSDOXA group at a dose of 10 ml/kg (2.48 g/mL) once daily for 28 days. ABX diluted in drinking water was administered to rats in the ABXOXA group from 14 days before OXA administration until the end of the study. The CON and OXA groups received saline. All rats were dissected on day 29 to collect fresh fecal samples, serum, plasma, DRG, and colon tissue samples. The flow charts of experimental procedures are shown in Fig. 1A and Additional file 1: Fig.S6-1A.

### Fecal microbiota transplantation (FMT)

ABX was administered to recipient rats (n = 12 per group) during 5 days before FMT [27]. Fresh stools from rats in the OXA, DSD, and DSDOXA groups were administered to OXA-treated recipient rats via FMT once daily for 28 days. Fecal samples were collected daily and mixed with tenfold saline in sterile tubes, and the resulting suspensions were centrifuged at 4000*g* for 5 min. Bacteria-enriched supernatants were immediately collected and orally administered to recipient rats at a dose of 10 mL/kg to avoid changes in bacterial components [28]. OIPN was induced in recipient rats as described above and sacrificed at day 29 to collect fresh fecal samples, serum, plasma, DRG, and colon tissue.

### Assessment of mechanical allodynia

Von Frey filaments (Aesthesio, DanMic Global, LLC, USA) were used to evaluate mechanical allodynia on treatment days 0, 7, 14, 21, and 28, as described before [29]. Rats were placed for about 10 min in a small cage with a mesh floor to acclimatize to the test. A vertical stimulus was focused on the plantar surface of the hind paw, to produce a constant force for 5 s [30]. When the rat manifested a positive response, such as withdrawing or licking the paw, the minimum force eliciting reproducible paw withdrawal or shaking was recorded. Touch responses to Von Frey filaments with varying bending forces were measured 5 consecutive times with 30 s stimulation intervals. Three or more positive reactions were regarded as mechanical pain sensitivity, with a maximum strength of 15 g. A persistent decrease in the mechanical threshold signaled the presence of neurotoxicity.

### DRG histology

Nissl’s staining was used to assess damage to DRG neurons. Lumbar (L4-L5) DRG tissues were fixed in 4% paraformaldehyde solution, embedded in paraffin, and sectioned at 4–5 μm before conventional Nissl’s staining. Stained sections were examined through a light microscope.

### Colon histology

Hematoxylin and eosin (H&E) staining was used to assess damage to colon tissue. Samples were fixed in 4% paraformaldehyde, embedded in paraffin, and sectioned at 4–5 μm before routine H&E processing. Stained sections were examined through light microscopy.

Immunohistochemistry (IHC) was used to assess the expression of tight junction-related proteins in paraformaldehyde-fixed, paraffin embedded colon tissue. Citrate buffer (pH 6.0) was used for antigen retrieval. Endogenous peroxidase was blocked by 3% H_2_O_2_ and non-specific antigens were blocked with serum. The sections were incubated with primary antibodies against Mucin 2 (MUC 2) (1:500; ab272692; Abcam, USA), Claudin-1 (1:100; 13050-1-AP; Proteintech, China), Occludin (1:200; ab216327), and ZO-1 (1:500, ab221547; Abcam, USA), followed by addition of HRP-conjugated antibodies (1:100, Baiqiandu Biological Tech., Wuhan, China). DAB was used as chromogen for signal detection, and images were acquired by light microscopy.

Transmission electron microscopy (TEM) was used to assess the ultrastructure of colon tissue. Samples were fixed in 4% glutaraldehyde, post-fixed in 1% osmic acid at 25 °C for 2 h, dehydrated with graded ethanol series, embedded in Epon 812, sectioned into ultrathin slices (60–80 nm), double-stained with uranium acetate and lead citrate, and finally examined by TEM.

### Enzyme-linked immunosorbent assay (ELISA)

ELISA kits for determination of LPS (MBE11054), IL-6 (MBE10288), and TNF-α (MBE10037) in serum and DRG extracts were obtained from Nanjing Mallbio Biotech. Co., Ltd. (Nanjing, China) and utilized based on the manufacturer’s instructions.

### Gene expression analysis

Expression levels of TNF-*a* and IL-6 mRNA were quantified by real-time PCR (qPCR) using total RNA extracted from DRG based on the instructions of the FastPure Cell/Tissue Total RNA Isolation Kit (Vazyme, Nanjing, China). RNA was reverse-transcribed to cDNA using a QuantiTect® Reverse Transcription Kit (Qiagen, CA, USA), and qPCR analysis implemented using a QuantiNova ™ SYBR® Green PCR Kit (Qiagen, CA, USA) on an AB StepOnePlus real-time PCR system (Applied Biosystems, Foster City, CA, USA). Sequences of primers for qPCR are shown in Additional file 1: Table S2. The expressions of target genes were normalized to GAPDH and calculated by the 2^−ΔΔCт^ algorithm.

### Sequencing analysis of 16S rDNA

At the end the experimental treatments (day 29) fresh rat feces were collected on dry ice and transferred to Shanghai Biotree Biotech Co., Ltd. (Shanghai, China) for 16S rDNA assays. The QIAamp Fast DNA Stool Mini Kit (Qiagen, CA, USA) was used to extract microbial DNA. The hypervariable V3–V4 region of the 16S rDNA sequence was targeted for PCR amplification, followed by purification and quantification. Purified amplicons were sequenced on an Illumina NovaSeq 6000 platform (Illumina, San Diego, CA, USA), and analyzed for microbial diversity.

### GC–MS-based SCFAs analysis

Fecal samples were softened with dH_2_O, homogenized, ultrasound-treated, and centrifuged. Supernatants were mixed with 0.1 mL 50% H_2_SO_4_ and 0.8 mL 2-methylvaleric acid (25 mg/mL stock in ethyl ether) as internal standard. Samples were placed on an oscillating shaker, ultrasonicated, and centrifuged, and the upper ether solution was subjected to GC–MS analysis on An Agilent 7890 gas chromatograph system and an Agilent 5975C mass spectrometer. Data were gathered in multiple reaction monitoring modes with characteristic fragment ions of SCFAs obtained by references. Quantitative measurements of SCFAs in feces were performed according to the data acquired and standard curves from references.

### UHPLC-QE-MS-based untargeted metabolomics analysis

Untargeted metabolomics analyses were performed on plasma samples by liquid chromatography-tandem mass spectrometry (LC–MS/MS). The UHPLC system (Vanquish, Thermo Fisher Scientific, MA, USA) with UPLC BEH Amide columns (2.1 mm × 100 mm, 1.7 µm) was connected to a Q Exactive HFX mass spectrometer (Orbitrap MS, Thermo Scientific, MA, USA). Detailed metabolite extraction, LC–MS/MS analysis, data preprocessing and annotation, and data analysis procedures were described in the Additional file 1: Material 3. The online analytical tool MetaboAnalyst (https://www.metaboanalyst.ca) was applied for analyzing pathway enrichment.

### Statistical analysis

All data are presented as mean ± standard error of the mean (SEM). Statistical analyses were conducted on GraphPad Prism (GraphPad Software, CA, USA). Differences between groups were analyzed by unpaired two-tailed Student’s t-test. P < 0.05 was considered significant. Correlation coefficients were assessed by Spearman’s correlation analysis.

## Results

### DSD alleviated OIPN by inhibiting DRG inflammation

In OXA group, OXA caused symptoms of body weight loss, decreased withdrawal threshold for mechanical allodynia, and neuronal cell injury (Fig. 1). DSD treatment improved OXA-induced body weight loss (Fig. 1B). As expected, mechanical pain thresholds were persistently decreased by OXA treatment (*P* < 0.01; Fig. 1C). Notably, by the end of treatments (day 28), mechanical pain thresholds after DSD treatment were significantly increased by 176% (*P* < 0.01), with no significant differences compared with the CON group (Fig. 1C). In line with these findings, DSD treatment significantly enhanced stained cytoplasm and increased shrunken nucleoli by 91%, improved OXA-induced DRG injury (*P* < 0.01) (Fig. 1D).

ELISA results showed that OXA treatment increased the expression of LPS, IL-6, and TNF-α in both serum and DRG, and that DSD treatment significantly reversed this effect (Fig. 1E). DSD significantly inhibited the expressions of LPS (20%), IL-6 (15%), and TNF-α (18%) in serum and LPS (25%), IL-6 (14%), and TNF-α (39%) in DRG comparing to OXA group. Gene expression analysis by qPCR showed that DSD remarkably attenuated the mRNA levels of IL-6 (23%) and TNF-α (59%) in DRG (Fig. 1F). These data support the conclusion that DSD protects against OIPN by inhibiting the inflammatory response in DRG.

### DSD protected against OXA-induced gut microbiota dysbiosis

**Fig. 2:**
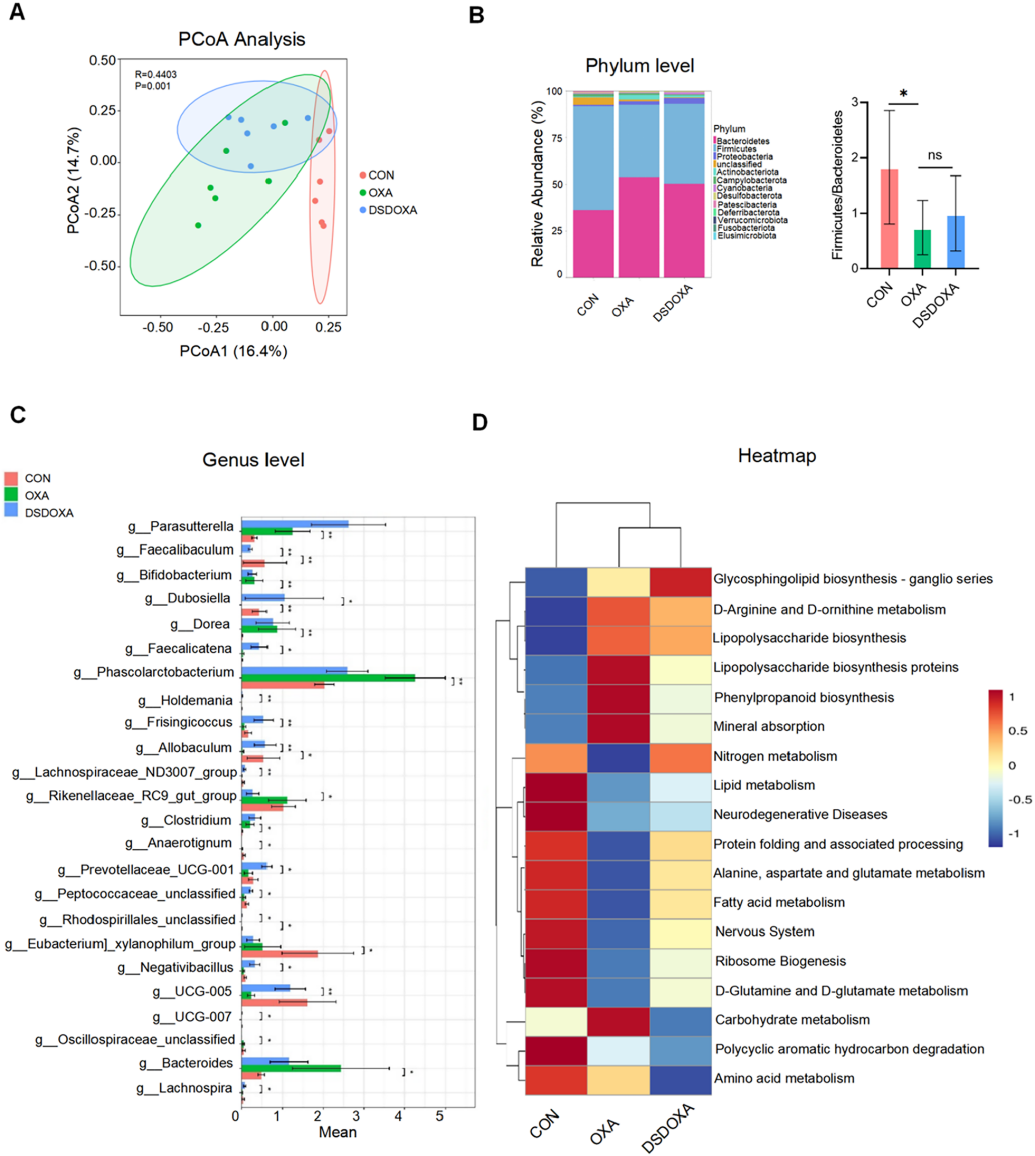
DSD protected against OXA-induced gut microbiota imbalance. **A** PCoA analysis of gut microbiota composition based on ASVs. **B** Phylum-level analysis of gut bacteria diversity among experimental groups. Histograms show *Firmicutes* to *Bacteroidetes* ratios. **C** Genus-level analysis of gut bacteria diversity among the experimental groups. **D** Heatmap of functional profiles of microbial communities in the CON, OXA, and DSDOXA groups. Gradient transition from blue to red reflects change in abundance, from low to high. *P < 0.05, **P < 0.01, vs. OXA group; n = 6 in every group

To assess the relationship between OIPN and specific alterations in the gut microbiota, we first examined microbiome diversity. The Chao1 and Shannon indexes were decreased after OXA administration, and DSD treatment reversed this trend (Additional file 1: Fig. S4). Principal coordinate analysis (PCoA) indicated obvious clustering of amplicon sequence variants (ASVs) abundance in the three groups (nanosim: *R* = 0.442387,* P* = 0.001, Fig. 2A). Variance analysis was next used to assess changes in gut microbiota related to DSD treatment. At the phylum level, the abundance of Firmicutes was decreased by 30%, while that of Bacteroidetes was increased by 58% after OXA treatment. The ratio of Firmicutes to Bacteroidetes was decreased by 60% after OXA treatment (*P* < 0.05). Treatment with DSD increased the ratio by 35%, but not significantly (Fig. 2B). Intergroup differences in bacterial composition were next assessed at the genus level (Fig. 2C). 11 genera were significantly different (6 were increased such as *Clostridium and Prevotella_9* and 5 were decreased such as *Faecalibaculum*, *Allobaculum*, *Rhodospirillales_unclassified*, and *Dubosiella*) after OXA administration. 17 genera were significantly different (14 were increased such as *Faecalibaculum*, *Allobaculum*, *Dubosiella*, *Rhodospirillales_unclassified*, *UCG-005*, *Prevotellaceae_UCG-001*, *Peptococcaceae_unclassified*, *UCG-007*, *Frisingicoccus*, *Holdemania*, *Faecalicatena*, and *Negativibacillus* and 3 were decreased including *Anaerotignum*, *Rikenellaceae_RC9_gut_group*, and *Oscillospiraceae_unclassified*) by DSD treatment. In particular, significantly increased abundance of four beneficial genera that were significantly downregulated by OXA treatment, namely *Faecalibaculum* (90 times), *Allobaculum* (19 times), *Dubosiella*, and *Rhodospirillales_unclassified*, was detected after DSD treatment.

Based on the above data, we next analyzed functional alterations associated with dysregulated gut microbiota in the OIPN rat model (Fig. 2D). Pathway enrichment analysis showed that multiple metabolic pathways, including ribosome biogenesis, carbohydrate metabolism, phenylpropanoid biosynthesis, protein folding and associated processing, mineral absorption, alanine, aspartate, and glutamate metabolism, nitrogen metabolism, d-arginine and d-ornithine metabolism, d-glutamine and d-glutamate metabolism, lipopolysaccharide biosynthesis proteins, lipopolysaccharide biosynthesis, lipid metabolism, and fatty acid metabolism were significantly impacted by OXA treatment. Notably, these metabolic alterations especially d-glutamine and d-glutamate metabolism, lipopolysaccharide biosynthesis, were attenuated or reversed after administration of DSD (Fig. 2D).

### DSD attenuated OXA-induced metabolic alterations

**Fig. 3:**
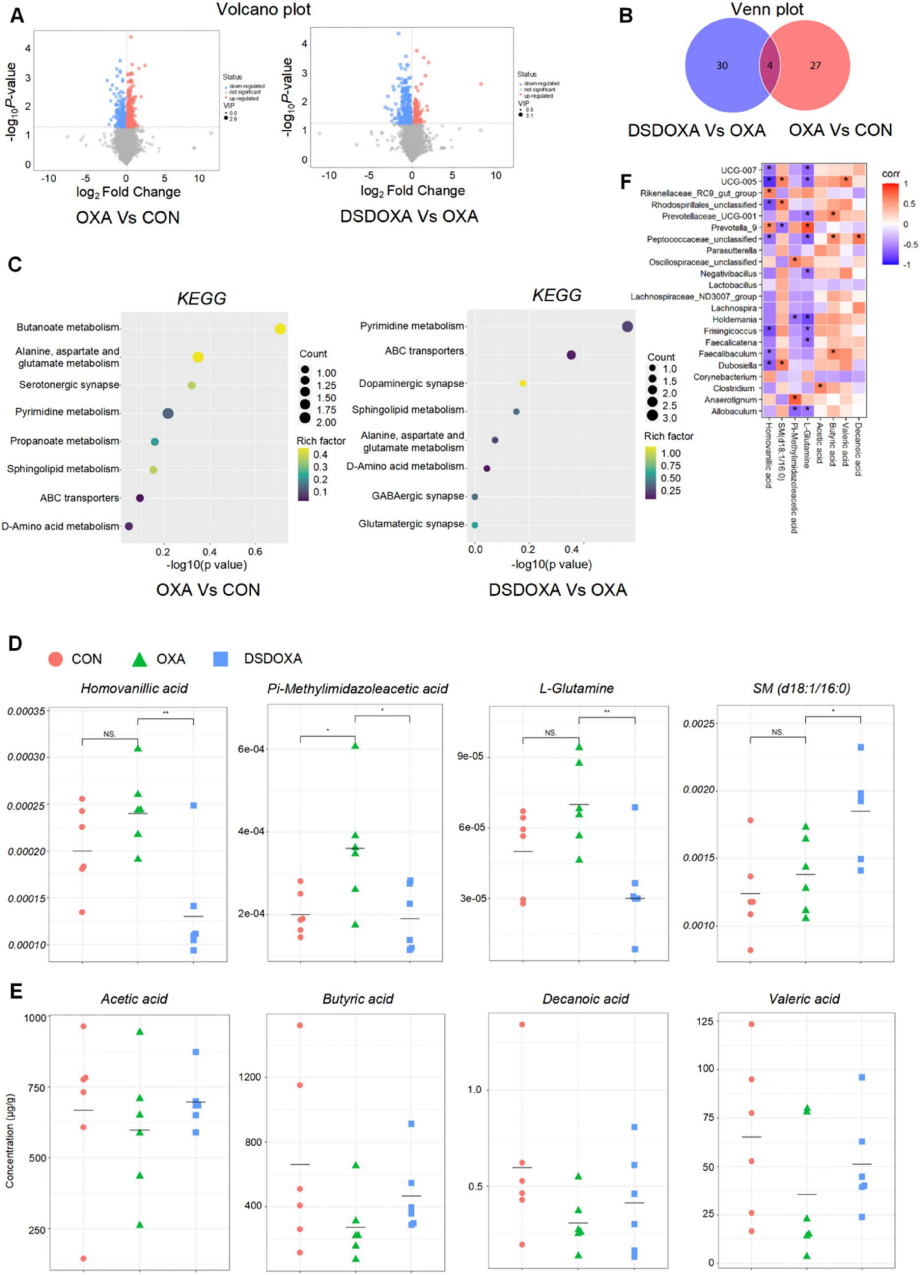
DSD attenuated OXA-induced metabolic alterations. **A** Volcano plots showing differential metabolites under two comparison strategies. **B** Venn plot showing overlapping differential metabolites under two comparison strategies. **C** Bubble plots showing the most relevant pathways co-enriched by the differential metabolites. **D** Relative abundance of critical and prominent secondary metabolites associated with the most relevant metabolic pathways in the three groups, expressed as relative increase. **E** GC–MS analysis of SCFAs (acetic acid, butyric acid, valeric acid, and decanoic acid) in feces. **F** Heatmap of Spearman’s correlation between DSD-adjusted gut microbiota and regulated metabolites. *P < 0.05, **P < 0.01, vs. OXA group; n = 6 in every group

To evaluate whether DSD treatment can counteract OXA-related metabolic alterations, we conducted untargeted metabolomics analyses in plasma. Volcano plots indicated a differential metabolite profile after both OXA and DSD treatments (Fig. 3A). Specifically, 31 differential metabolites (20 upregulated and 11 downregulated ones) were recorded after OXA treatment compared to the CON group. In turn, 34 differential metabolites (20 downregulated and 14 upregulated ones) were determined by DSD administration (Additional file 1: Table S5-1 and S5-2). Venn diagram analyses showed four differential metabolites in common among those two groups (Fig. 3B). Under two different comparison strategies, the most relevant pathways co-enriched by the four shared differential metabolites included pyrimidine metabolism, sphingolipid metabolism, alanine, aspartate and glutamate metabolism, ABC transporters, and d-amino acid metabolism (Fig. 3C). DSD significantly adjusted the abundance of the four critical metabolites in all the co-enriched pathways as well as in other three pathways, namely glutamatergic synapse, GABAergic synapse, and dopaminergic synapse (*P* < 0.05). Specifically, after DSD treatment, pi-methylimidazoleacetic acid (neurotoxins), homovanillic acid, and l-glutamine (neurotransmitters) were decreased, whereas sphingomyelin (d18:1/16:0) (neuroprotective agents) was increased (*P* < 0.05, Fig. 3D). These data demonstrated that OIPN is associated with significant changes in plasma metabolome, which can be counteracted by treatment with DSD.

Given that the pro-proliferative effects of DSD on SCFA-producing bacteria, we employed GC–MS based targeted metabolomics to evaluate fecal levels of SCFAs. Results showed that DSD treatment increased the OXA-induced decrease of acetic acid, butyric acid, valeric acid, and decanoic acid (Fig. 3E).

### DSD-recovered gut microbiota was closely related to metabolism

Spearman’s correlation analysis was used to assess potential correlations between gut microbiota composition and metabolite abundance after DSD treatment (Fig. 3F). Acetic acid, butyric acid, valeric acid, and decanoic acid contents were positively correlated with *Prevotellaceae_UCG-*001, *Faecalibaculum*, *Clostridium*, *UCG-*005, and *Peptococcaceae_unclassified*. Homovanillic acid levels were positively correlated with *Prevotella*_9, and while negatively correlated with *Faecalibaculum*, UCG-005, *Dubosiella*, *Rhodospirillales_unclassified*, and *Peptococcaceae_unclassified*. Sphingomyelin (d18:1/16:0) showed a positive correlation with *Dubosiella*, *Rhodospirillales_unclassified*, and *UCG-*005, and displayed a negative correlation with *Prevotella*_9. Pi-methylimidazoleacetic acid levels were positively correlated with unclassified *Oscillospiraceae* and *Anaerotignum*, and negatively correlated with *Holdemania* and *Allobaculum*. l-glutamine was positively correlated with *Prevotella*_9, and showed a notably negative correlation with *UCG-*005, *Holdemania*, *Allobaculum*, *Frisingicoccus*, *Prevotellaceae*_*UCG*-001, *Negativibacillus*, *Faecalicatena*, *Peptococcaceae_unclassified*, and *UCG*-007.

### DSD attenuated the OXA-induced increase in intestinal permeability

**Fig. 4:**
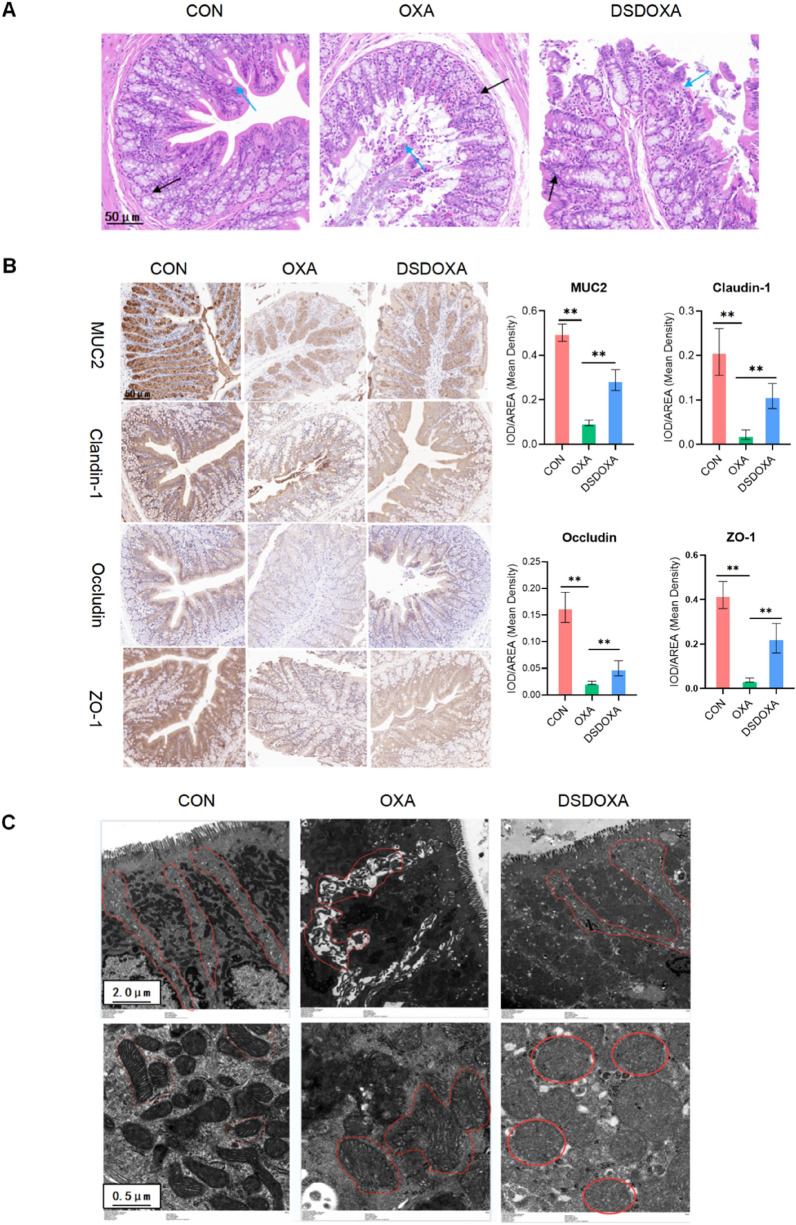
DSD attenuated OXA-induced intestinal permeability. **A** Representative images of colon sections stained with H&E (20 ×). Epithelial cells (blue arrows) and the mucosal layer (black arrow) are indicated. **B** Representative IHC images showing the expression of tight junction-related proteins in colon tissues (20 ×). Histograms show IOD/ARE (Mean density). **C** Transmission electron microscopy (2K × , 10K ×) analysis of epithelial junction complex and bridge and mitochondria in colon tissue. *P < 0.05, **P < 0.01, vs. OXA group; n = 3 in every group

As shown in Fig. 4A, DSD recovered the disruption of tissue morphology including detachment of epithelial cells in the mucosal layer, exposure of the lamina propria, and edema of submucosa in OXA-treated rats. Interestingly, no inflammation was observed in both OXA and DSD-treated rats. IHC revealed DSD treatment increased the lower expression of MUC 2 (201%), Claudin-1 (395%), Occludin (117%), and ZO-1 (495%) proteins in OXA-treated rats (*P* < 0.01, Fig. 4B). Moreover, results of TEM illustrated DSD treatment restored the damage of junction complexes and bridge, widened intercellular gaps, and swollen, disarranged, and blurred mitochondrial ridges in colon samples from OXA-treated rats (Fig. 4C).

### OPIN was prevented by antibiotic intervention

Gut microbiota depletion by antibiotic cocktail (ABX) intervention was used to evaluate the potential role of the gut microbiota in OIPN (Additional file 1: Fig. S6-1 and S6-2). We found that after ABX intervention OXA treatment did not significantly decrease body weight, but reduced mechanical pain thresholds, DRG injury and DRG inflammation as well as dysfunction of intestinal tight junction-related proteins, and ultrastructural changes in the colon. The importance of the gut microbiota in OIPN is thus highlighted by the reduced neurotoxicity and intestinal permeability exhibited by our pseudo-sterile rat model.

### FMT transferred the protective effects of DSD on OIPN

**Fig. 5:**
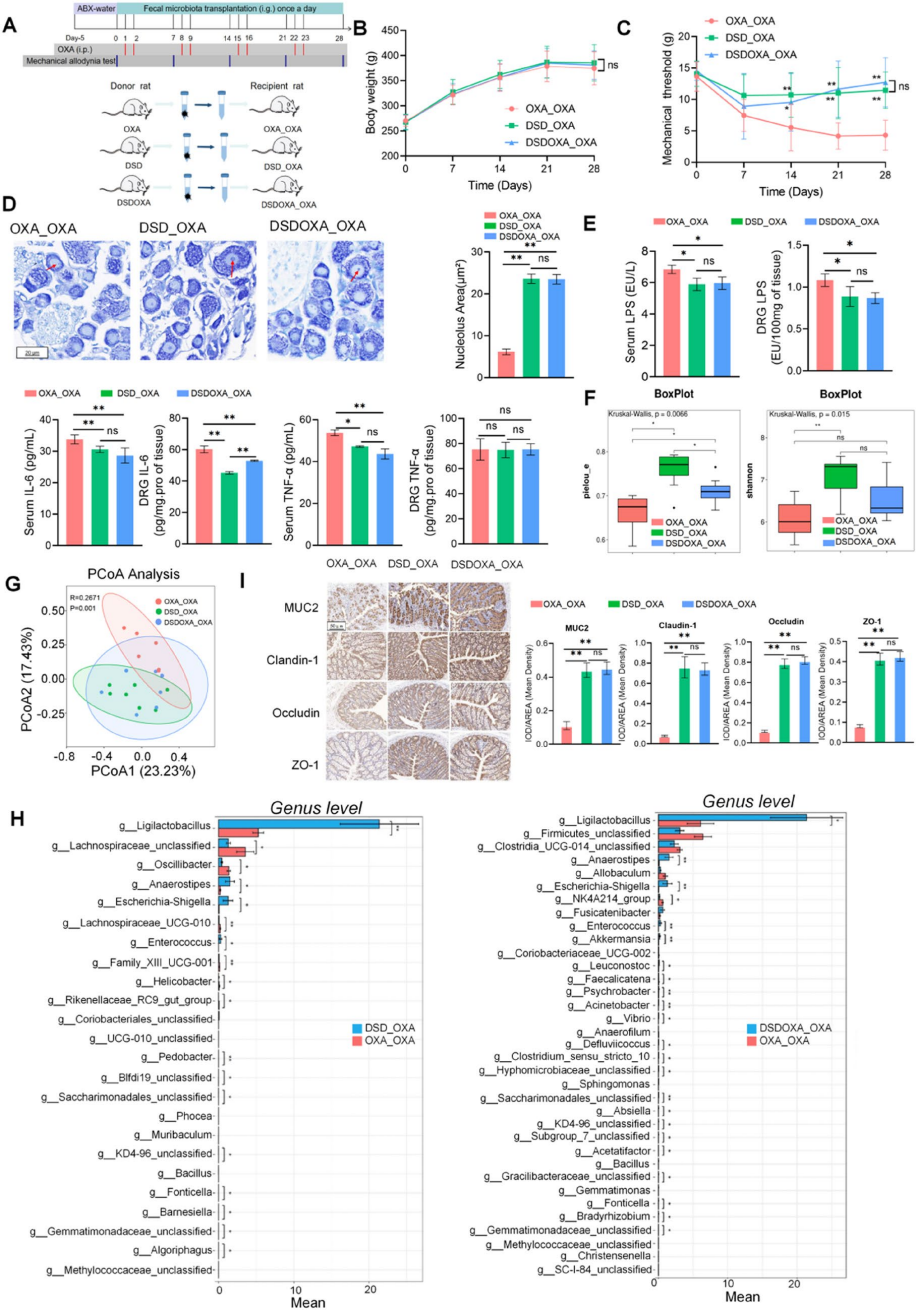
The protective effects of DSD in OIPN are transferred by FMT. **A** FMT protocol. **B** Body weight measurements. **C** Measurement of withdrawal threshold for mechanical allodynia (von Frey test). **D** Representative images of Nissl-stained DRG sections (63 ×). Lightly stained cytoplasm and shrunken nucleoli (red arrow) were analyzed. Histograms show nucleolar area. **E** ELISA-based detection of LPS, IL-6, and TNF-α in serum and DRG extracts. **F** Alpha-diversity of gut microbiota composition. **G** PCoA analysis of gut microbiota composition. **H** Genus-level analysis of bacterial composition under two comparison strategies. **I** Representative IHC images of colon sections showing the expression of tight junction-related proteins (20 ×). Histograms show IOD/ARE (mean density). *P < 0.05, **P < 0.01, vs. OXA_OXA group; n = 6 in OXA_OXA group, n = 7 in DSD_OXA and DSDOXA_OXA groups

To confirm that the protective effects of DSD on OIPN were mediated by gut microbiota, fecal samples from OXA-treated, DSD-treated, and DSDOXA-treated donor rats were transferred daily, over 28 days, to OXA-treated recipient rats (Fig. 5A). At the end of the intervention (day 28), there were no significant differences in body weight between DSD and DSDOXA receivers (DSD_OXA, DSDOXA_OXA groups) and OXA receivers (OXA_OXA group) (Fig. 5B). Interestingly, mechanical pain thresholds after FMT from either DSD- or DSDOXA- treated rats were significantly increased by 166% and 195% (P < 0.01) (Fig. 5C). In addition, Nissl’s staining on DRG showed that shrunken nucleoli were significantly increased in the DSD_OXA (284%) and DSDOXA_OXA (282%) groups compared with the OXA_OXA group (P < 0.01) (Fig. 5D). ELISA further showed that FMT from either DSD- and DSDOXA-treated donors remarkably downregulated the levels of LPS (13%, 14%) and IL6 (9%, 15%) in serum, levels of LPS (18%, 20%) and IL6 (25%, 12%) in DRG and levels of TNF-α (12%, 19%) in serum (P < 0.01) (Fig. 5E). Of note, there were no significant differences in paw withdrawal threshold, extent of DRG injury, and changes in LPS, IL6, and TNF-α between the DSD_OXA and the DSDOXA_OXA groups. These data strongly suggested that the beneficial effect of DSD on OXA-mediated DRG inflammation and OIPN is largely mediated by changes in gut microbiota composition.

Gut microbiota analysis suggested that Pielou_e and Shannon indexes were increased in the DSD_OXA (15%, 8%) and DSDOXA_OXA (16%, 8%) groups compared with the OXA_OXA group (P < 0.05) (Fig. 5F). A distinct clustering of ASVs abundance was observed for the OXA_OXA, DSD_OXA, and DSDOXA_OXA groups (nanosim: R = 0.2671, P = 0.001, Fig. 5G). In Fig. 5H, there were 24 differentially abundant bacteria between the DSD_OXA and the OXA_OXA groups and 35 differentially abundant bacteria between the DSDOXA_OXA and the OXA_OXA groups. Further analysis showed that a common decrease in the abundance of 10 bacterial groups, namely *Anaerostipes* (84%, 93%), *Saccharimonadale*s*_unclassified* (89%, 100%), *Escherichia-Shigella* (93%, 96%), *KD4-96_*(*Cloroflexi*)*_unclassified* (100%, 100%), *Ligilactobacillus* (75%, 72%), *Bacillus* (100%, 100%), *Enterococcus* (99%, 100%), *Fonticella* (95%, 100%), *Gemmatimonadaceae_unclassified* (100%, 100%), and *Methylococcaceae_unclassified* (100%, 100%), occurred in the DSD_OXA and the DSDOXA_OXA groups. These data indicated that FMT from DSD-treated normal or model rats protects against OIPN by restoring the diversity of gut microbiota.

IHC analysis revealed that colonic MUC 2 (300%, 309%), Claudin-1 (850%, 825%), Occludin (558%, 583%), and ZO-1 (409%, 425%) expression levels were significantly increased in the DSD_OXA and DSDOXA_OXA groups compared to the OXA_OXA group (P < 0.01) (Fig. 5I). These findings further indicated that DSD alleviates OIPN by restoring intestinal barrier dysfunction and gut microbiota homeostasis.

## Discussion

We found OXA could regulate the intestinal integrity and permeability, disorder gut microbiota and inflammatory metabolite, and ultimately result in OIPN, which is corresponding to previous research [31–35]. OIPN is caused with the existence of gut microbiota [31], which could also be demonstrated in this study by feeding ABX-water before and during OXA treatment. Moreover, DSD treatment could significantly alleviate OIPN by improving gut microbiota homeostasis and metabolism disorder, increasing colon tissue integrity as well as reducing intestinal permeability.

Our results found that the α-diversity of gut microbiota was increased by DSD treatment. Specifically, the abundance of *Bacteroidetes* was increased and the ratio of *Firmicutes* to *Bacteroidetes* was reduced, which may explain the decrease of LPS induced by DSD treatment [36]. Research suggested LPS mediates an inflammatory response in DRG by activating macrophages and TLR4 signaling [11, 15]. An improved epithelial barrier function reduced the leakage of LPS into the enterohepatic circulation, and then attenuated the inflammatory response in DRG neurons. Besides, the abundance of *Faecalibaculum*, *Allobaculum*, *Dubosiella*, and *Rhodospirillales_unclassified*, SCFA-producing bacteria, was also significantly upregulated by DSD. Thereby, the levels of SCFAs (especially acetic acid, butyric acid, valeric acid, and decanoic acid) were raised by DSD treatment in OIPN model. SCFAs have been reported to maintain microecological stability, enhance the mechanical barrier function of the intestinal mucosa, and inhibite intestinal permeability [37, 38].Taken together, DSD plays a protective role in OIPN through modulating gut microbiota composition, as well as affecting LPS and SCFAs production.

A clinical study suggests that OIPN may be treated by oral glutamine [39].In addition, animal experiments show that OIPN is relieved by N-methyl-D-aspartate receptor (NMDAR) inhibitors [40, 41]. An increase in glutamate concentration in the cerebrospinal fluid of the lumbar spinal cord, downregulation of glutamate transporter-1 (GLT-1) expression, and activation of NMDAR have been described in the OIPN model [42]. Thus, changes in glutamate metabolism pathway are crucially involved in OIPN, in association with peripheral nervous system hyperexcitability and release of pro-inflammatory factors [43]. l-glutamate can be converted into d-glutamate, a constituent of the peptidoglycan cell wall in most gram-negative bacteria, by glutamate racemase from Lactobacillus [44]. After d-glutamine is formed from d-glutamate by synthetase, it is transported into the extracellular fluid, taken up by neurons, and converted back into glutamate by a deaminase [45]. The levels of extracellular glutamate are decreased through this process, which may lead to neuronal excitotoxicity [46]. Our findings showed that DSD treatment enriched glutamate metabolism pathways. After DSD treatment, low abundance of Bacteroides, which encompasses important gram-negative bacteria, and high abundance of *Lactobacillus* should lead to further reduction in l-glutamate metabolism and increased d-glutamate metabolism. This may be indeed consistent with the decrease of l-glutamine after DSD treatment, which could be associated with decreased release of proinflammatory factors and reduced nociception. However, the specific bacterial genes affecting glutamate synthesis remain incompletely defined, and whether glutamate metabolism may be affected by differential regulation of GLT-1 and NMDAR expression is still unknown.

**Fig. 6:**
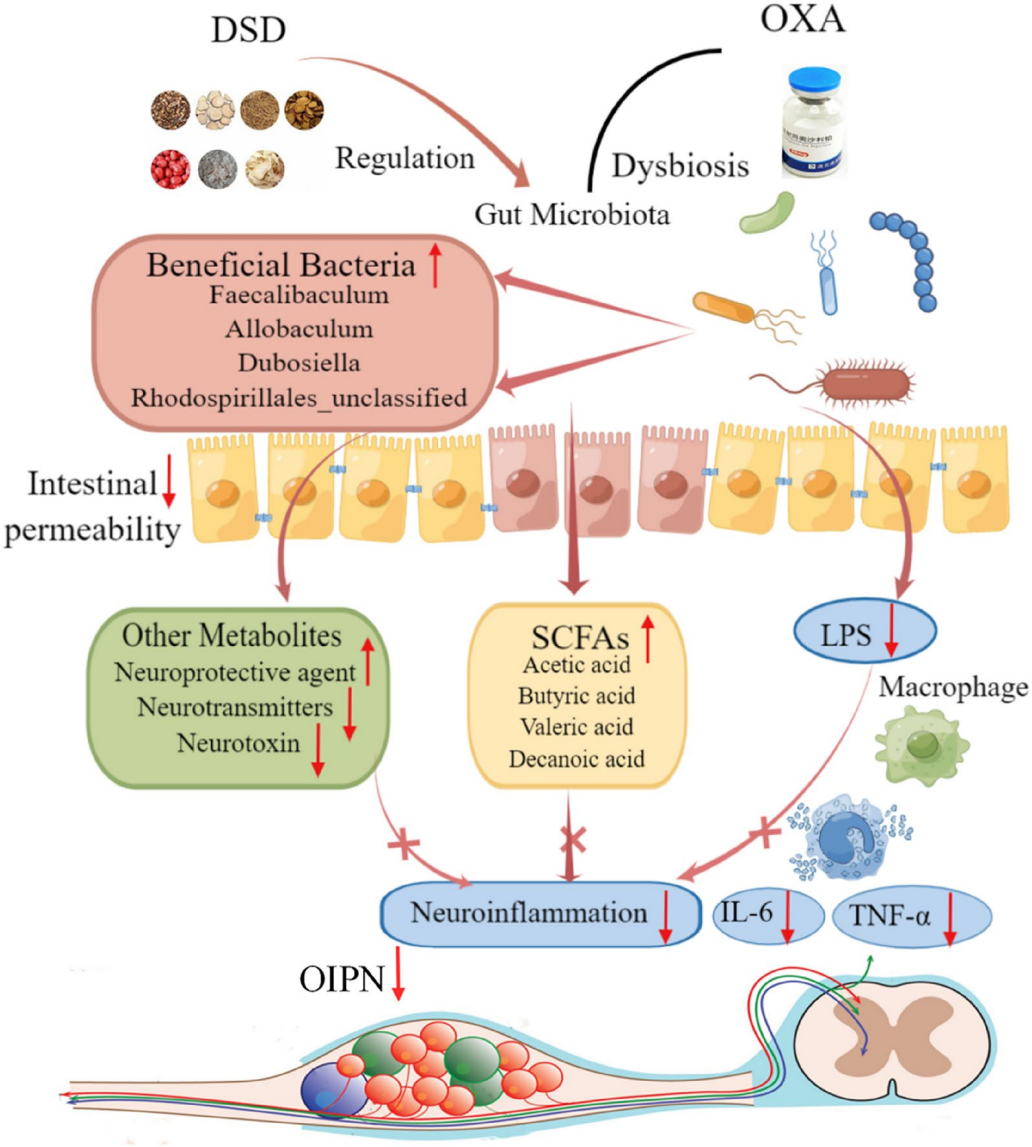
Schematic illustration of the possible mechanisms that DSD-induced changes in gut microbiota ameliorate OIPN symptoms. DSD treatment beneficially regulated OXA-induced gut dysbiosis by enriching beneficial bacteria such as *Faecalibaculum*, *Allobaculum*, *Dubosiella*, and *Rhodospirillales_unclassified*. This lead to reduced intestinal permeability, decreased LPS leakage, increased SCFA levels, decreased neurotransmitters and neurotoxins, increased neuroprotective agents, alleviating DRG inflammation and hyperalgesia

FMT has gradually arisen as effective strategy to treat diseases mediated by gut microbiota imbalances [13, 32]. In our study, after FMT from donors treated with DSD with or without concomitant OXA treatment, DRG inflammation and intestinal permeability of rats were significantly reduced, while gut microbiota diversity was significantly improved. These findings indicated that DSD confers protective effects on OIPN by regulating gut microbiota, mainly via enriching *Faecalibaculum*, *Allobaculum*, *Dubosiella*, and *Rhodospirillales_unclassified*, then reducing intestinal permeability and LPS production, increasing SCFA levels and neuroprotective agent, decreasing neurotransmitters and neurotoxin, as well as reducing neuroinflammation and hyperalgesia. A schematic diagram of the hypothetical mechanisms of DSD-induced changes in gut microbiota and ameliorated OIPN symptoms is presented in Fig. 6. DSD also plays an anti-inflammatory role in RA, mainly by affecting intestinal microbiome and its metabolites [1]. Moreover, similar to our results, glutamate metabolism, acetic acid, butyric acid, and valeric acid may also play a key role in the anti-inflammatory mechanism of DSD in RA. However, further research is needed to determine whether these metabolites or LPS through which metabolic pathways play a role in the formation of OIPN.

## Conclusion

In summary, this study showed that DSD effectively improves DRG inflammation, gut microbiota homeostasis, intestinal permeability and systemic metabolism in OIPN model. Correlation analysis revealed that the composition and abundance of gut microbiota are closely related to inflammation related metabolism. FMT demonstrated that DSD exerts therapeutic effects against OIPN by regulating gut microbiota diversity. All the results suggested that DSD prevents OIPN by alleviating gut microbiota dysbiosis and thus potentially regulating the neuroinflammation related metabolic disorder. The outcomes of study provide a scientific basis for the clinical application of DSD for the treatment of OIPN, and confirme the gut microbiota as a relevant therapeutic target of OIPN.

### Supplementary Information

**Additional file 1: Fig. S1.** UPLC-QTOF-MS/MS Analysis of BPI plots of DSD. **Table S1.** UPLC-QTOF-MS/MS constituents identification information table of DSD. **Table S2.** Sequences of primers for qPCR. Material 3. Supplementary materials of the untargeted Plasma metabolomic analysis. **Fig. S4.** Chao1 and Shannon index. **Table S5-1.** Different metabolites identifified between the OXA and CON groups. **Table S5-2.** Different metabolites identifified between the DSDOXA and OXA groups. **Fig. S6-1.** ABX alleviated OIPN and the inflammatory response. **Fig. S6-2.** ABX attenuated OXA-induced intestinal permeability.

## Data Availability

The datasets used and analyzed during the current study are available from the corresponding author on reasonable request.

## Abbreviations

OXA: Oxaliplatin
OIPN: Oxaliplatin-induced peripheral neuropathy
DSD: Danggui Sini decoction
DRG: Dorsal root ganglia
LPS: Lipopolysaccharide
SCFAs: Short-chain fatty acids
TLR4: Toll-like receptor 4
GABA: Gamma aminobutyric acid
MUC 2: Mucin 2
ZO-1: Zonula occludens-1
NMDAR: n-methyl-d-aspartate receptor
GLT-1: Glutamate transporter-1
