# Supplementary material for: Danggui Sini decoction alleviates oxaliplatin-induced peripheral neuropathy by regulating gut microbiota and potentially relieving neuroinflammation related metabolic disorder
Source: Chin Med. 2024 Apr 7;19:58. doi: 10.1186/s13020-024-00929-7 (PMC10999090; doi:10.1186/s13020-024-00929-7)

Additional file

**1 Chemical characterization of DSD**

The major chemical constituents in DSD formula granules were qualitatively characterized by targeted chemomics methods we previously established. As shown in Fig S1 and Table S1,Nine constituents, namely Albiflorin、Paeoniflorin、Liquiritin apioside、Liquiritin、Galloylpaeoniflorin or Galloylalbiflroin or their isomers、Glyyunnanprosapogenin、Glycyrrhizic acid、Uralsaponin B、Ligustilide, were determined by UPLC-QTOF-MS/MS mode（Table S1）.In positive ion mode, six constituents were determined; in negative ion mode, eight constituents were determined（Fig S1）.


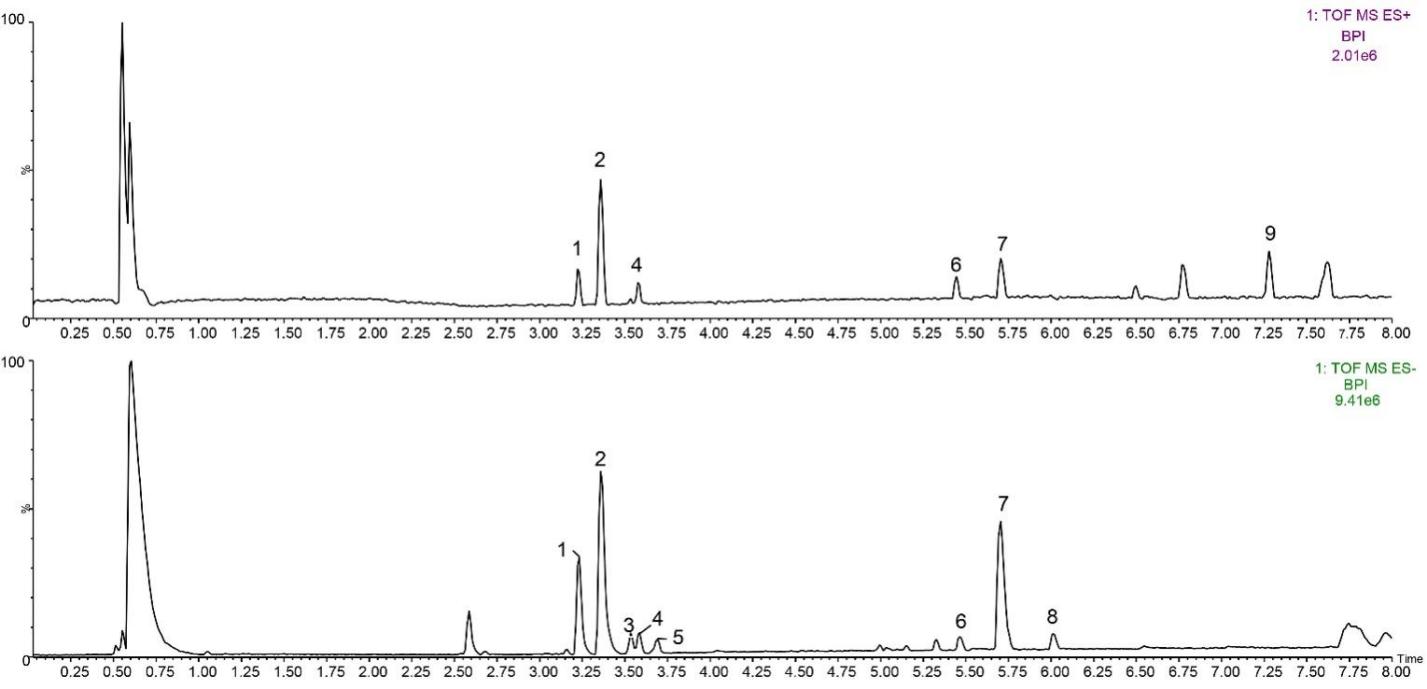


**Fig S1 UPLC-QTOF-MS/MS Analysis of BPI plots of DSD**

（1- Albiflorin；2- Paeoniflorin；3- Liquiritin apioside；4- Liquiritin；5- Galloylpaeoniflorin or Galloylalbiflroin or their isomers；6- Glyyunnanprosapogenin D；7- Glycyrrhizic acid；8- Uralsaponin B；9- Ligustilide）

**Table S1 UPLC-QTOF-MS/MS constituents identification information table of DSD**

| **Peak no.** | **t_R_**  **(min)** | **Identity** | **Molecular**  **formula** | **Proposed**  **adduct ions or fragment ions** | ***m*/*z***  **(Da)** | **Mass accuracy**  **(ppm)** |
| --- | --- | --- | --- | --- | --- | --- |
| 1 | 3.23 | Albiflorin | C_23_H_28_O_11_ | [M–H+HCOOH]^−^  [M–H]^−^ | 525.1626  479.1555 | 3.4  0.2 |
| 2 | 3.36 | Paeoniflorin | C_23_H_28_O_11_ | [M–H+HCOOH]^−^  [M–H]^−^  [M–H–CHO]^−^ | 525.1608  479.1536  449.1451 | 0.0  2.1  0.7 |
| 3 | 3.53 | Liquiritin apioside | C_26_H_30_O_13_ | [M–H]^−^  [M–Api]^−^  [M–Api–Glc]^−^ | 549.1620  417.1180  255.0650 | 2.2  -1.4  -2.7 |
| 4 | 3.59 | Liquiritin | C_21_H_22_O_9_ | [M–H]^−^  [M–H–Glc]^−^ | 417.1180  255.0652 | -1.4  -2.0 |
| 5 | 3.69 | Galloylpaeoniflorin or  Galloylalbiflroin or their isomers | C_30_H_32_O_15_ | [M–H]^−^ | 631.1664 | 0.2 |
| 6 | 5.46 | Glyyunnanprosapogenin D | C_42_H_62_O_17_ | [M–H]^−^  [M–H–CO_2_]^−^ | 837.3942  793.4008 | 3.9  -0.3 |
| 7 | 5.70 | Glycyrrhizic acid | C_42_H_62_O_16_ | [M–H]^−^  [M–H–GlcA]^−^  [M–H–2GlcA]^−^ | 821.3960  645.3650  469.3324 | 3.5  1.7  1.3 |
| 8 | 6.02 | Uralsaponin B | C_42_H_62_O_16_ | [M–H]^−^  [M–H–GlcA]^−^ | 821.3984  645.3651 | 2.9  1.9 |
| 9 | 7.28 | Ligustilide | C_12_H_14_O_2_ | [M+H]^+^ | 191.1071 | -0.5 |

# 2 Additional file for gene Sequences

**Table S2 Sequences of primers for qPCR**

| Gene | Primer direction | Sequence |
| --- | --- | --- |
| GAPDH | Forward | 5′-CATCAAGAAGGTGGTGAAGC-3′ |
|  | Reverse | 5′- CCTGTTGCTGTAGCCGTATT-3′ |
| IL-6 | Forward | 5′-ATATACCACTTCACAAGTCGG-3′ |
|  | Reverse | 5′-GGCAAATTTCCTGGTTATATCC-3′ |
| TNF-*a* | Forward | 5′-ACCACGCTCTTCTGTCTACTG-3′ |
|  | Reverse | 5′-CTTGGTGGTTTGCTACGAC-3′ |

# 3 Additional file material of the untargeted Plasma metabolomic analysis

**Metabolites Extraction**

100 μl of Plasma sample was weighted to an EP tube, and 400 μL extract solution (methanol: acetonitrile = 1: 1, with isotopically-labeled internal standard mixture) was added. Then the samples were mixed by a vortex for 30 s and sonicated for 10 min in an ice water bath. The homogenization and sonication cycle was repeated for 3 times. Then the samples were incubated for 1 h at -40°C and centrifuged at 12000 rpm for 15 min at 4°C. The resulting supernatant was transferred to a fresh glass vial for analysis. The quality control (QC) sample was prepared by mixing an equal aliquot of the supernatants from all of the samples.

**LC-MS/MS Analysis**

The liquid chromatography-tandem mass spectrometry (LC-MS/MS) analyses were performed using a UHPLC system (Vanquish, Thermo Fisher Scientific) with a Ultra-high performance liquid chromatography (UPLC) BEH Amide column (2.1 mm × 100 mm, 1.7 μm) coupled to Q Exactive HFX mass spectrometer (Orbitrap MS, Thermo). The mobile phase consisted of 25 mmol/L ammonium acetate and 25 ammonia hydroxide in water (pH = 9.75) (A) and acetonitrile (B). The auto-sampler temperature was 4°C, and the injection volume was 3μL. The QE HFX mass spectrometer was used for its ability to acquire MS/MS spectra on information-dependent acquisition (IDA) mode in control of acquisition software (Xcalibur, Thermo). In this mode, the acquisition software continuously evaluates the full scan MS spectrum. The ESI source conditions were set as following: sheath gas flow rate as 30 Arb, Aux gas flow rate as 25 Arb, capillary temperature 350°C, full MS resolution as 60000, MS/MS resolution as 7500, collision energy as 10/30/60 in NCE mode, spray Voltage as 3.6 kV (positive) or -3.2 kV (negative), respectively.

**Data preprocessing and annotation**

The raw data were converted to the mzXML format using ProteoWizard and processed with an in-house program, which was developed using R and based on XCMS, for peak detection, extraction, alignment, and integration. Then an in-house MS2 database (BiotreeDB) was applied in metabolite annotation. The cutoff for annotation was set at 0.3. For metabolites with the same annotation in positive and negative mode, we keep those with larger peaks and reject those with smaller peaks.

**Metaboanalyst** (https://www.metaboanalyst.ca) was carried out for data cleaning, statistical analysis, and pathway enrichment analysis. The peak intensity matrix with a zero value in more than 50% of samples was filtered by removing peaks. The remaining missing values were replaced by one-fifth of the minimum positive value of each variable. Deviating values are filtered if their relative standard deviation is >25% and normalized using the mean value. Orthogonal projections to latent structures discriminant analysis (OPLS-DA) algorithm, fold change (fc), and *t*-tests were adopted to identify the metabolites with significant differences between groups. The permutation test was performed to validate the OPLS-DA model. Differential expressed metabolites (DEMs) were identified by strict criteria, namely, variable importance in the projection (VIP) value > 1, log2 (fc) > |2|, *p* < 0.05. The Kyoto Encyclopedia of Genes and Genomes (KEGG) pathways of DEMs were enriched by Metaboanalyst and presented as potential targets with a threshold of *p* < 0.05.

# 4 α-diversity of gut microbiota

# Chao1 and Shannon index were used to assess the diversity of gut microbiota by 16S rDNA gene sequencing of fecal samples. As is showed in Fig S4, the two indexes were all decreased in OXA group, but DSD reversed the trend (P＞0.05).


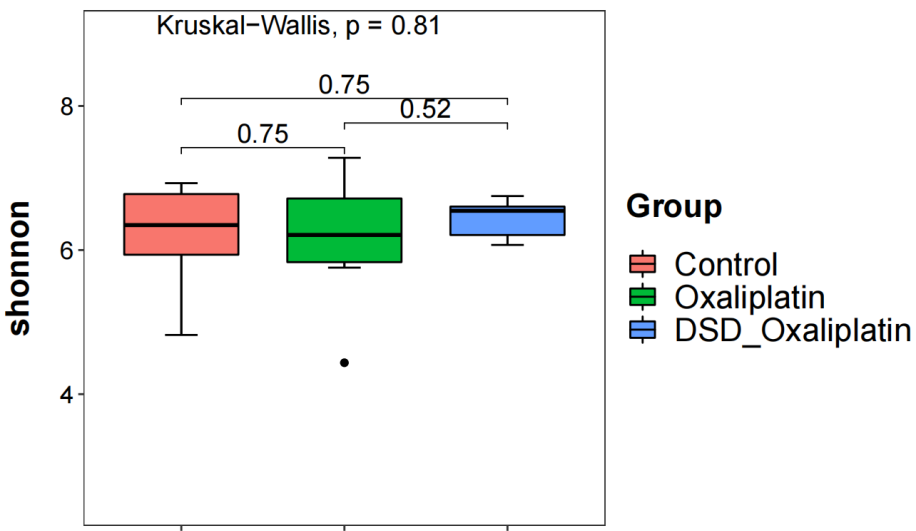

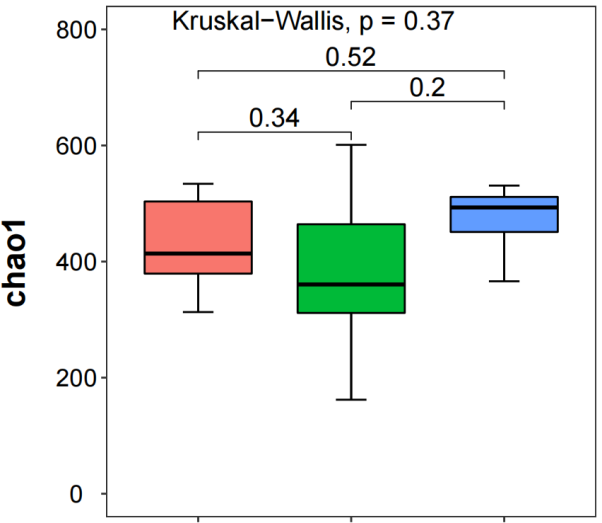


**CON**

**OXA**

**DSDOXA**

**Fig S4** **Chao1 and Shannon index（n=6）**

**5** **Differential metabolites derived from untargeted metabolomics analysis**

There were 31 different metabolites identified between the OXA and CON groups, among which 20 increased and 11 reduced in the OXA group. There were 34 different metabolites adjusted by DSD treatment, among which 20 downregulated and 14 upregulated compared with the OXA group as is showed in Table. S5-1 and S5-2.

**Table S5-1 Different metabolites identified between the OXA and CON groups**

| **id** | **MS2 name** | **MS2 score** | **MEAN OXA** | **MEAN CON** |
| --- | --- | --- | --- | --- |
| 3 | Pyruvic acid | 1 | 0.001633187 | 0.001333893 |
| 21 | Deoxycytidine | 0.999595308 | 0.000219429 | 0.000198736 |
| 25 | L-Gulose | 0.999504462 | 0.001590148 | 0.001363785 |
| 40 | Glycerol tripropanoate | 0.997958385 | 3.01698E-06 | 5.00165E-06 |
| 51 | Ethyl oleate | 0.996172077 | 0.000386281 | 0.000537796 |
| 71 | 2-Hydroxyethanesulfonate | 0.991279077 | 0.000591514 | 0.001307142 |
| 99 | 2-Methyl-6-(1-propenyl)pyrazine | 0.985076462 | 5.56668E-05 | 7.24948E-05 |
| 140 | Peperinic acid | 0.968795154 | 2.78897E-05 | 4.54389E-05 |
| 147 | L-Lactic acid | 0.966363615 | 0.00145818 | 0.001284057 |
| 158 | Pyrimidine | 0.961689923 | 1.22246E-06 | 1.7733E-06 |
| 171 | Cholesterol | 0.949235846 | 0.000220903 | 0.000142838 |
| 194 | Tetradecanedioic acid | 0.934093923 | 6.17281E-06 | 1.35318E-05 |
| 223 | Succinic acid semialdehyde | 0.911719308 | 0.001214357 | 0.001058645 |
| 288 | Orotic acid | 0.852807 | 0.000118062 | 6.81331E-05 |
| 300 | apo-[3-methylcrotonoyl-CoA:carbon-dioxide ligase (ADP-forming)] | 0.839545 | 2.3029E-05 | 4.41635E-05 |
| 320 | 4-Hydroxy-1H-indole-3-acetonitrile | 0.810167308 | 3.56541E-05 | 1.64979E-05 |
| 356 | 1-Deoxy-D-xylulose 5-phosphate | 0.761485538 | 0.000880296 | 0.000728862 |
| 367 | Ascorbic acid | 0.746691846 | 0.001811155 | 0.001154946 |
| 391 | 2-Acetylpyrazine | 0.712134615 | 7.85865E-05 | 3.72655E-05 |
| 400 | Pi-Methylimidazoleacetic acid | 0.705098692 | 0.000356733 | 0.000202566 |
| 409 | PC(20:4(5Z,8Z,11Z,14Z)/14:0) | 0.683603 | 2.5383E-05 | 3.49858E-05 |
| 503 | PI(20:2(11Z,14Z)/18:2(9Z,12Z)) | 0.573717077 | 1.99994E-05 | 1.83389E-05 |
| 519 | SM(d18:1/20:0) | 0.560242154 | 5.84987E-06 | 4.91157E-06 |
| 552 | 2-[(Methylthio)methyl]-2-butenal | 0.525037923 | 2.11969E-05 | 2.78404E-05 |
| 563 | Etonogestrel | 0.511369385 | 1.08755E-06 | 8.85377E-07 |
| 570 | DG(20:5(5Z,8Z,11Z,14Z,17Z)/18:2(9Z,12Z)/0:0) | 0.494578231 | 1.49329E-05 | 2.74381E-05 |
| 576 | Ethyl methyl trisulfide | 0.481744269 | 1.11369E-05 | 9.14795E-06 |
| 588 | 5,6-DHET | 0.445900308 | 0.000114509 | 9.80734E-05 |
| 602 | PE(P-18:1(9Z)/20:3(5Z,8Z,11Z)) | 0.416363769 | 3.34157E-05 | 2.74959E-05 |
| 605 | Threoninyl-Aspartate | 0.411337462 | 6.08909E-06 | 5.14343E-06 |
| 614 | PE(P-18:1(11Z)/18:3(6Z,9Z,12Z)) | 0.359374846 | 1.72898E-05 | 1.39678E-05 |

**Table S5-2 Different metabolites identified between the DSDOXA and OXA groups**

| **id** | **MS2 name** | **MS2 score** | **MEAN DSDOXA** | **MEAN OXA** |
| --- | --- | --- | --- | --- |
| 7 | Cytosine | 0.999886308 | 0.002016181 | 0.002248652 |
| 21 | Deoxycytidine | 0.999595308 | 0.000194367 | 0.000219429 |
| 79 | Caprylic acid | 0.990293385 | 0.000176003 | 0.000234612 |
| 98 | Cotinine N-oxide | 0.985100538 | 3.20325E-06 | 5.70989E-06 |
| 118 | N,N'-Diacetylhydrazine | 0.978316615 | 6.35654E-06 | 1.1822E-05 |
| 150 | N-Acetyl-L-phenylalanine | 0.965473385 | 2.30054E-05 | 1.2018E-05 |
| 193 | Vinylacetylglycine | 0.934955385 | 1.56125E-05 | 2.85508E-05 |
| 218 | Dimethylethanolamine | 0.915608 | 4.91651E-06 | 3.50103E-06 |
| 225 | L-Pipecolic acid | 0.910170308 | 6.53025E-06 | 3.4901E-06 |
| 238 | Homovanillic acid | 0.899900385 | 0.000134876 | 0.000244197 |
| 261 | Grandisine III | 0.885227923 | 3.19914E-06 | 4.52251E-06 |
| 275 | N-Acryloylglycine | 0.870247154 | 0.000706766 | 0.000507562 |
| 285 | 8,15-DiHETE | 0.857585692 | 9.04066E-06 | 1.84371E-05 |
| 312 | Valyl-Valine | 0.816322923 | 1.96495E-06 | 1.26586E-06 |
| 336 | SM(d18:1/16:0) | 0.784547385 | 0.001849297 | 0.001376148 |
| 359 | Koeniginequinone A | 0.758035462 | 1.47605E-05 | 2.11956E-05 |
| 380 | (R)-lipoic acid | 0.725154231 | 0.000169039 | 0.000131108 |
| 384 | Na,Na-Dimethylhistamine | 0.720006308 | 0.000103713 | 0.000186949 |
| 391 | 2-Acetylpyrazine | 0.712134615 | 3.4639E-05 | 7.85865E-05 |
| 394 | Oxoglutaric acid | 0.708959923 | 2.32642E-05 | 1.67111E-05 |
| 399 | N-Acetylhistamine | 0.705202231 | 8.27121E-06 | 2.44387E-05 |
| 400 | Pi-Methylimidazoleacetic acid | 0.705098692 | 0.000192653 | 0.000356733 |
| 414 | 6-Deoxyfagomine | 0.678764615 | 7.58319E-06 | 1.13444E-05 |
| 418 | Methoxyeugenol | 0.676285154 | 1.76373E-05 | 3.23734E-05 |
| 445 | gamma-Glutamylisoleucine | 0.638543 | 1.55266E-05 | 7.09791E-06 |
| 475 | SM(d18:1/22:0) | 0.594571692 | 2.5018E-05 | 1.80499E-05 |
| 485 | SM(d18:1/14:0) | 0.589604769 | 1.34421E-05 | 1.05768E-05 |
| 534 | PC(22:6(4Z,7Z,10Z,13Z,16Z,19Z)/20:5(5Z,8Z,11Z,14Z,17Z)) | 0.548796308 | 3.30523E-06 | 4.41594E-06 |
| 542 | Isoleucyl-Histidine | 0.540102846 | 1.5173E-06 | 3.65969E-06 |
| 552 | 2-[(Methylthio)methyl]-2-butenal | 0.525037923 | 3.12977E-05 | 2.11969E-05 |
| 578 | 4-(Methylnitrosamino)-1-(3-pyridyl)-1-butanol | 0.470532692 | 4.3792E-06 | 7.40053E-06 |
| 580 | L-Glutamine | 0.465690923 | 3.40867E-05 | 6.96965E-05 |
| 589 | Cortisone | 0.439404192 | 1.49239E-05 | 2.43323E-05 |
| 609 | L-Histidine trimethylbetaine | 0.390231 | 1.52912E-06 | 4.02802E-06 |

**6** OPIN was prevented by antibiotic intervention

Gut microbiota depletion by antibiotic cocktail (ABX) treatment were used to evaluate the potential role of the gut microbiota in OIPN (Supplementary Fig. S6-1 and S6-2).

**Figure legends**

**Fig. S6-1.** ABX alleviated OIPN and the inflammatory response. **A)** Protocols of drug administration and mechanical allodynia test, in the rat OIPN model. **B)** Body weight measurements. **C)** Measurement of withdrawal threshold for mechanical allodynia (von Frey test). **D)** Representative images of Nissl-stained DRG sections (63×). Lightly stained cytoplasm and shrunken nucleoli (red arrow) were analyzed. Histograms showed nucleolar area. **E)** ELISA-based detection of LPS, IL-6, and TNF-α in serum and DRG extracts. **P* <0.05, ***P* <0.01, vs. OXA group; n=11 in the CON group; n=10 in the OXA groups, n=9 in the ABXOXA group.

**Fig. S6-2.** ABX attenuated OXA-induced intestinal permeability. **A)** Representative images of colon sections stained with H&E (20×). Epithelial cells (blue arrows) and the mucosal layer (black arrow) are indicated. **B)** Representative IHC images showing the expression of tight junction-related proteins in colon tissues (20×). Histograms show IOD/ARE (Mean density). **C)** Transmission electron microscopy (2K×, 10K×) analysis of epithelial junction complex and bridge and mitochondria in colon tissue. **P* <0.05, ***P* <0.01, vs. OXA group; n=3 in every group.


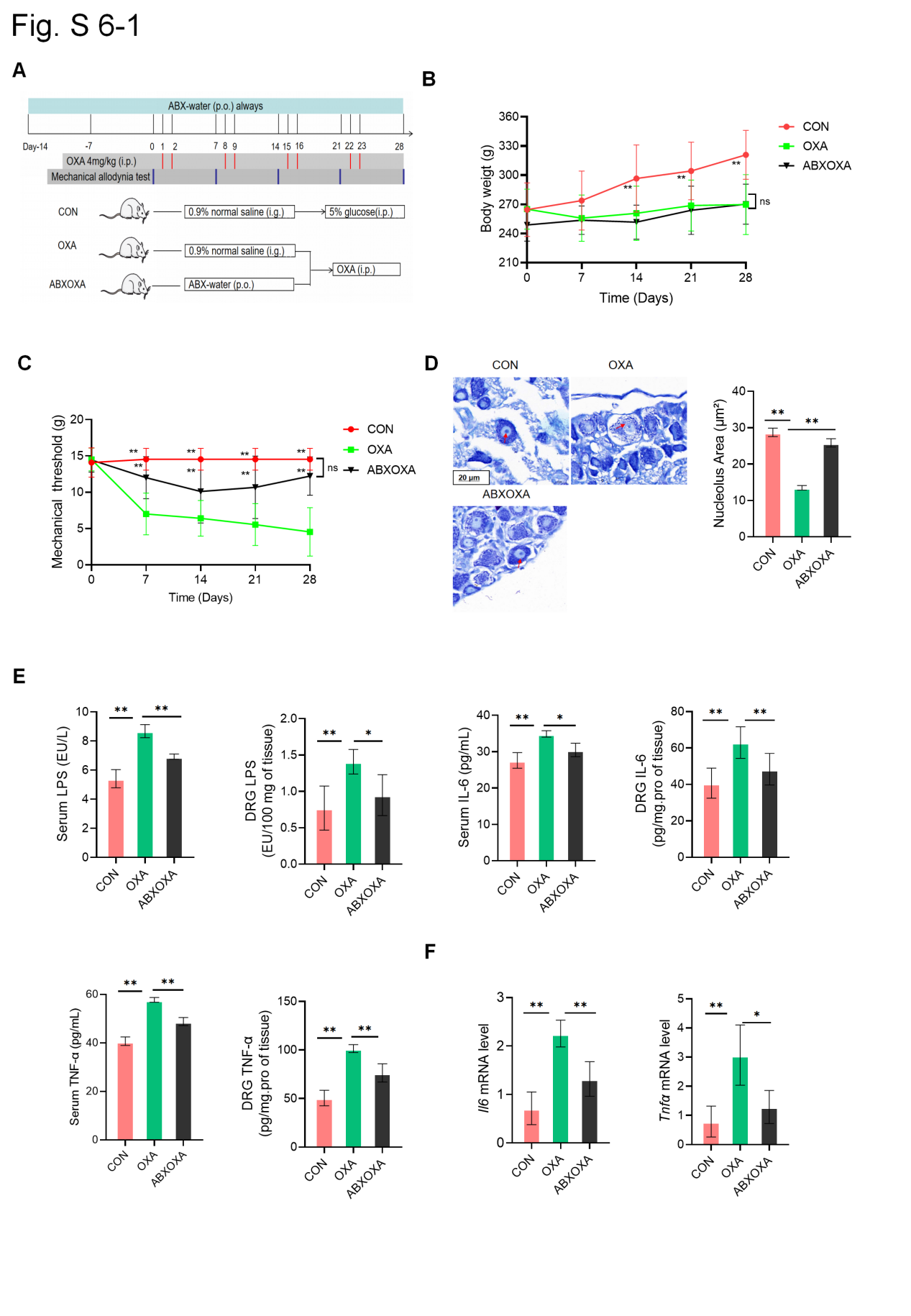

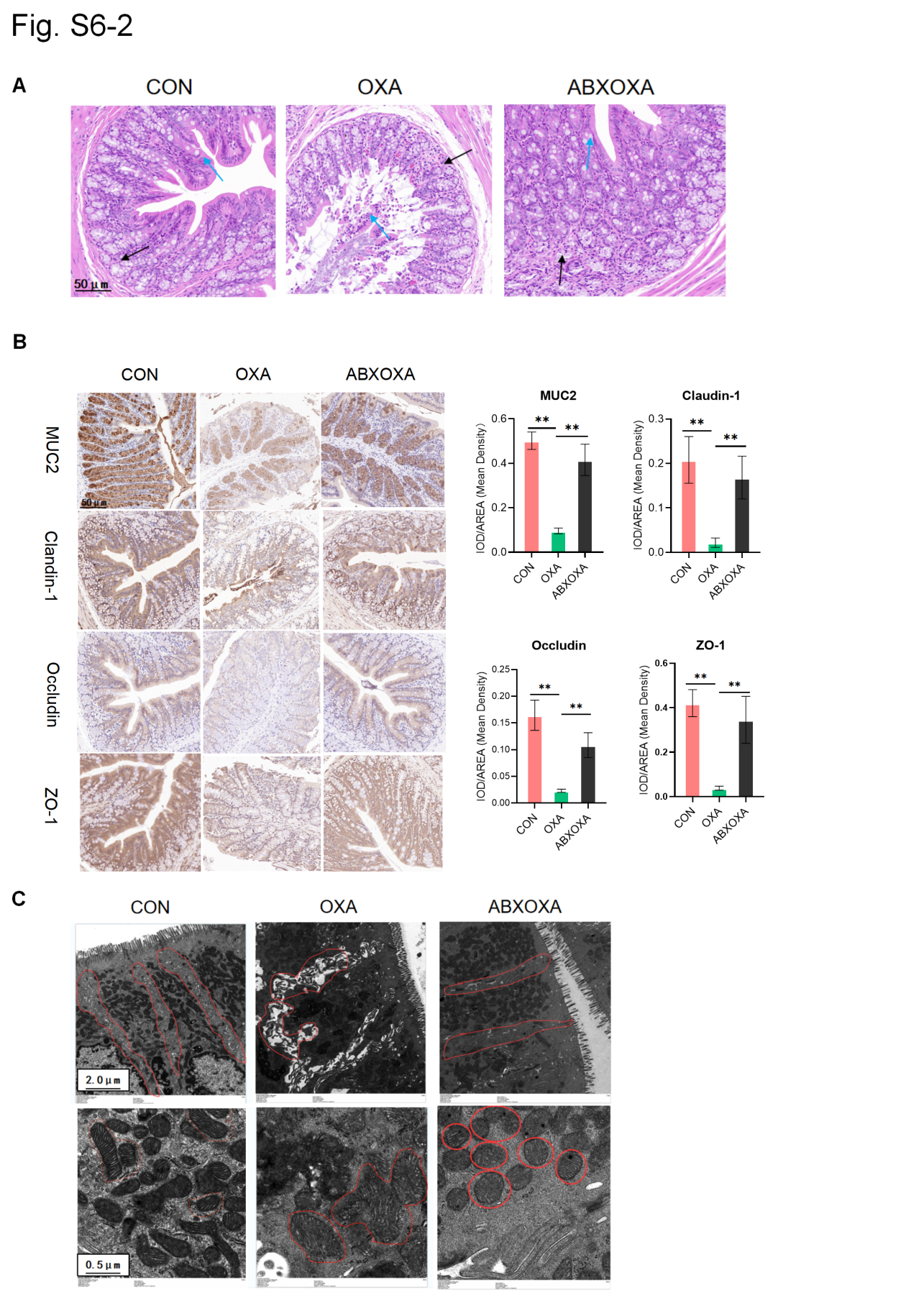

Supplement: Supplementary file 1 — Additional file 1: Fig. S1. UPLC-QTOF-MS/MS Analysis of BPI plots of DSD. Table S1. UPLC-QTOF-MS/MS constituents identification information table of DSD. Table S2. Sequences of primers for qPCR. Material 3. Supplementary materials of the untargeted Plasma metabolomic analysis. Fig. S4. Chao1 and Shannon index. Table S5-1. Different metabolites identifified between the OXA and CON groups. Table S5-2. Different metabolites identifified between the DSDOXA and OXA groups. Fig. S6-1. ABX alleviated OIPN and the inflammatory response. Fig. S6-2. ABX attenuated OXA-induced intestinal permeability. [file 13020_2024_929_MOESM1_ESM.docx]
